# Antiphospholipid Syndrome in a Pregnant Female Presenting with Severe Thrombocytopenia and Bleeding

**DOI:** 10.1155/2015/234878

**Published:** 2015-02-05

**Authors:** Kunal Mahajan, Virender Katyal, Suvrat Arya, Meha Shrama

**Affiliations:** ^1^Department of Medicine, PGIMS Rohtak, Haryana 124001, India; ^2^Department of Medicine, KGMC, Lucknow 226008, India

## Abstract

The antiphospholipid antibody syndrome (APS) is defined by the persistent presence of antiphospholipid antibodies in patients with recurrent venous or arterial thromboembolism or pregnancy morbidity. Antithrombotic therapy is the mainstay of treatment given the high risk of recurrent thromboembolism that characterizes this condition. Despite the prothrombotic nature of APS, thrombocytopenia is present in a proportion of patients, which can complicate management and limit the use of antithrombotic therapy. The mechanism of APS-associated thrombocytopenia is multifactorial and its relation to thrombotic risk is poorly characterized. The presence of thrombocytopenia does not appear to reduce thrombotic risk in patients with APS, who can develop thromboembolic complications necessitating antithrombotic treatment. In these cases, treatment of the thrombocytopenia may be necessary to facilitate administration of antithrombotic agents. We present such a pregnant lady with history of recurrent pregnancy losses who presented with severe thrombocytopenia and bleeding manifestations, who was subsequently diagnosed to have antiphospholipid antibody syndrome. She was initially managed with steroids and when her platelet counts improved, antithrombotic therapy was started. She delivered an uneventful and successful pregnancy outcome without any complications during follow-up.

## 1. Case History

A 30-year-old housewife with past history of 4 unsuccessful pregnancies presented with thirteen weeks of amenorrhoea (fifth pregnancy) and recurrent low grade fever of 15-day duration. She had noted gums bleeds and petechial spots all over the body since 7 days. There was no history of bleeding per vaginum, hematemesis, melena, or epistaxis. She denied any arthralgias, myalgias, oral ulcers, red eyes, weight loss, pain abdomen, migraine, or constitutional symptoms. No similar complaints were present in the past. Patient had history of three second trimester abortions previously and 1 pregnancy loss at 3-month-period gestation which was not evaluated. Her family history was noncontributory. She weighed 46 kg and examination was significant for pallor, nonpalpable petechial spots over the trunk and limbs, stable vitals, with a palpable uterus over the pelvic brim corresponding to 12-week gestation.

Laboratory investigations showed platelet count—20,000/cumm, haemoglobin—6 gms %, Hematocrit—18.7% with RBC indices revealing microcytic hypochromic anemia (MCV—68 fl and MCH—26.9 pg) and total leucocyte count (TLC) of 6000/cumm with granulocytes 68%, lymphocytes 28%. She had normal liver and kidney function tests. Her bleeding profile showed persistently decreased platelet count 20,000/cumm with prolonged bleeding time and normal clotting time, with normal prothrombin time (PT) and activated partial thromboplastin time (aPTT). Her human immunodeficiency virus (HIV), hepatitis-B surface antigen (HBsAg), and anti-hepatitis-C (HCV) antibodies were negative. Her dengue serology and malaria parasite study were negative. Fibrin degradation products (FDP) and D-dimer study were found to be negative. She was investigated for antiphospholipid antibody (APLA) syndrome in view of recurrent pregnancy losses and IgM anti-cardiolipin antibody was found to be positive (48 MPL units, that is, >99th percentile). Antibodies against beta 2 glycoprotein 1 and lupus anticoagulant were negative. Her anti-nuclear antibody (ANA) by enzyme linked immunoassay and immunofluorescence assay was negative. Anti-double strand- (ds) DNA antibody study was also negative. Bone marrow aspiration examination revealed normal marrow cellularity and slightly increased megakaryocytes. Ultrasonography (USG) abdomen confirmed single live fetus of 12 weeks.

Considering severe thrombocytopenia and bleeding diathesis, the patient was given platelet concentrates. She was also given packed cells for anemia and prednisolone 60 mg/day was started for immunosuppression in order to raise the platelet count. Patient showed considerable improvement with treatment and her platelet counts improved to 50,000 three days after starting steroid therapy. When the platelet count increased to 1 lac/cumm at 7th day of starting steroid therapy, a maintenance dose of 60 mg/day of prednisolone was continued and gradually tapered over 2-month time. Her platelet count had become normal in 2-month time and measured 1,60,000/cumm. IgM anticardiolipin antibody was repeated after 12 weeks and it came out to be positive (42 MPL units > 99th percentile). In view of previous recurrent pregnancy loses and positive anticardiolipin antibodies on two separate occasions 12 weeks apart, a diagnosis of APL syndrome was made, presenting during pregnancy with severe thrombocytopenia with bleeding complications. In view of the previous pregnancy losses and corrected platelet count, patient was then started on tablet Ecosprin 75 mg once daily and unfractionated heparin 5000 U s/c twice daily. Patient was kept on regular follow-up since then, with regular supervised obstetric evaluations and regular monitoring of platelet count. Neither did she have any new haemorrhagic episodes nor was there any evidence of thrombotic complications over the entire follow-up. Her pregnancy remained uneventful after that and she delivered a healthy baby at 37th week of gestation by normal vaginal delivery.

## 2. Discussion

Diagnosis of APLA syndrome is based on clinical criteria of thromboembolism or pregnancy morbidity and laboratory findings of raised titres of antiphospholipid antibodies that are present on two or more occasions at least 12 weeks apart [1]. Although isolated rise of IgM anticardiolipin antibodies has been least associated with APLA syndrome, it is still compatible with the SYDNEY criteria set for diagnosis [1]. APLA syndrome predominantly presents as a prothrombotic disorder. It is one of few conditions that can appear with both arterial and venous thromboembolism. Thrombocytopenia is reported in about 20 to 40 percent of patients with APLA syndrome and is usually mild (70–120 × 10^9^/L) and does not require clinical intervention. Severe thrombocytopenia (platelet count < 50 × 10^9^/L) may be seen in 5 to 10 percent of patients [2, 3]. Interestingly, the presence of thrombocytopenia in patients with APLA syndrome is not typically associated with haemorrhagic complications [3, 4]. In our case severe thrombocytopenia was present (20 × 10^9^/L) along with bleeding manifestations. The differential diagnosis in patients with APS presenting with thrombocytopenia includes thrombotic thrombocytopenic purpura (TTP), heparin induced thrombocytopenia (HIT), and disseminated intravascular coagulation (DIC). Antiphospholipid antibodies can be present in TTP and other thrombotic microangiopathies including haemolytic uraemic syndrome and HELLP (hemolysis, elevated liver enzymes, low platelets) syndrome [5]; however presence of microangiopathic haemolytic anemia with schistocytes on peripheral blood smear and evidence of hemolysis differentiates these syndromes from APLA syndrome. Measurement of ADAMTS-13 can also be done to document TTP.

For HIT, history of use of heparin is essential. In DIC patients generally have thrombocytopenia and thrombotic and hemorrhagic events. There is derangement of bleeding time, clotting time, international normalized ratio (INR), and thrombocytopenia in the setting of a compatible clinical picture of precipitating DIC. Our patient had no evidence of haemolytic anemia and had normal FDP and D-dimer levels and no heparin was used in the past.

There are likely different mechanisms resulting in thrombocytopenia in patients with APLA syndrome. Many patients are initially diagnosed with idiopathic thrombocytopenic purpura (ITP), and autoantibodies directed against platelet glycoproteins have been detected in patients with antiphospholipid antibodies [6] as well as in patients with APLA syndrome [7]. Thrombocytopenia in these patients may be due to immune-mediated clearance of platelets, as in ITP. However, the frequent finding of thrombocytopenia and thrombosis in patients with APLA syndrome suggests that antiphospholipid antibodies interact with platelets in a manner that triggers platelet aggregation and thrombosis. In vitro and in vivo studies have demonstrated that antiphospholipid antibodies can bind platelets, increasing platelet aggregation and activation in the presence of subthreshold concentrations of thrombin, adenosine diphosphate, or collagen [8]. The intracellular events following platelet binding remain incompletely elucidated, but activation of arachidonic acid and thromboxane production and expression of glycoprotein IIb/IIIa have all been shown to occur following antiphospholipid antibody binding to platelets [9]. Thrombocytopenia was a clinical criterion used to define the syndrome in the initial classification of APLA syndrome; it was not included in the most recently proposed classification [1]. Another issue of clinical importance in evaluating thrombocytopenia associated with APLA syndrome is the risk for future development of thrombosis. In one study in APLA syndrome patients were divided into three groups according to platelet counts as normal, moderately thrombocytopenic (50–100 × 10^9^/L), or severely thrombocytopenic (<50 × 10^9^/L); the rates of future thrombosis were 40 percent, 32 percent, and 9 percent, respectively [3]. These data show that moderate thrombocytopenia does not prevent thrombosis in patients with APLA syndrome. Antithrombotic prophylaxis should be considered in these patients [2, 3]. In our study, patient had severe thrombocytopenia and once her platelet count improved we started her on antithrombotic prophylaxis.

Treatment of APLA syndrome associated thrombocytopenia may be required to facilitate antithrombotic treatment and minimize bleeding complications. There are no trials evaluating optimal management in this setting, and no guidelines exist regarding when treatment is required and what treatments should be used.

In general, treatment of APLA syndrome associated thrombocytopenia is indicated in overt bleeding irrespective of platelet counts. Also patients with APLA syndrome have thromboembolic complications despite being thrombocytopenic and require treatment to raise the platelet count to at least 30–50 × 10^9^/L before antithrombotic therapy can be administered. Treatment of APLA syndrome associated thrombocytopenia is similar to that of ITP. Treatment options include glucocorticoids, intravenous immunoglobulin and immunosuppressive agents (azathioprine and cyclophosphamide), and off-label use of newer agents such as rituximab. Pregnant women with APLA syndrome and thrombocytopenia often require treatment to facilitate use of epidural anesthesia and to ensure adequate haemostasis at the time of delivery. In these cases, intravenous immunoglobulins and early delivery, if possible, are preferred [10].

Our case was a pregnant lady with APLA syndrome with bad obstetric history and severe thrombocytopenia with bleeding in current conception. She responded well to steroid therapy with good recovery of platelet count. Patient was successfully started on antithrombotic treatment and delivered a healthy baby at term.

Since there are no well defined guidelines for management of these complicated cases, criteria for treatment of thrombocytopenia and initiation of antithrombotic therapy in APLA syndrome patients with thrombocytopenia need further studies to elucidate clear guidelines. Till then decisions must be individualized in light of patient-specific risk factors and preferences.
